# Economy in Cocaine

**Published:** 1900-12-15

**Authors:** 


### ECONOMY IN COCAINE.

Dr. Wyatt Wingrave,[1] physician to the Central London Throat and Ear Hospital, says that, in view of the high price of cocaine and the occasional intolerance to it and its salts manifested by certain people, he has carried out some experiments to see whether it would be possible to reduce the quantity employed and increase its efficacy when used in ear, throat, and nose work. As a result it appears that a 2 per cent. solution of the alkaloid in equal parts of almond and petroleum oil proved an efficient local anæsthetic for the examination of and for slight operations upon the nose and throat. This solution may be applied either by an atomiser or a cotton-wool swab, and it is found that, although the oily preparation takes a little longer to act, the resulting anæsthesia is more prolonged than that produced by a watery solution. With regard to the salt it was found that a watery solution containing 5 per cent. of hydrochlorate of cocaine and 2 per cent. of sulphate of soda was distinctly more rapid than, and produced an anæsthesia quite as complete as that caused by much stronger solutions without the addition of the soda salt. Apparently the addition of the sulphate of soda increased the penetrative power of the solution. Anyhow, economy in such an expensive drug as cocaine is a distinct advantage to the pocket, besides the advantage to such patients as are especially susceptible to it of having as small a quantity used as will produce the desired effect.

[1] Lancet, Dec. 8.

